# A bibliometric analysis of income and cardiovascular disease: Status, Hotspots, Trends and Outlook

**DOI:** 10.1097/MD.0000000000021828

**Published:** 2020-08-21

**Authors:** Ye Ding, Dingwan Chen, Xufen Ding, Guan Wang, Yuehua Wan, Qing Shen

**Affiliations:** a School of Public Health, Hangzhou Medical College; b Institute of Information Resources, Zhejiang University of Technology; c Library, Zhejiang University of Technology, Hangzhou, China.

**Keywords:** cardiovascular disease, income, bibliometric, elderly, prevention

## Abstract

**Background:**

Income is 1 of the socio-economic indicators and could directly influence the health outcomes of cardiovascular disease (CVD). The relationship between income and CVD has attracted more and more scholars’ attention in the past 20 years.

**Methods:**

To study the current research dynamics of this field, a bibliometric analysis was conducted to evaluate the publications from 1990 to 2018 based on the Science Citation Index Expanded database. By using the Derwent Date Analyzer software, the following aspects were explored:

(1) historical trend of the topic;
(2) the main contributor: leading countries, leading institutions, leading research areas and journals;
(3) representative authors;
(4) most frequently used keywords;
(5) most cited papers.

**Results:**

The USA ranked first in this field, followed by UK and Canada in terms of number of publications. As for institutions, Harvard University took the leading place in the number of publications, as well as the h-index. Plos One had the most publications and “health” was the most frequent used keyword. The leading research area was “public environmental occupational health”.

**Conclusions:**

In conclusion, the elderly, the children and the puerpera were the main study population in this field and “disease prevention” was the main study direction. The most concerned health issues in this field were “obesity” and “diet”. There might be a lack of articles that explore the associations between income and CVD with a global perspective. Articles on this content are urgently warranted.

## 1 Introduction

Non-communicable diseases are reaching epidemic proportions worldwide ^[1]^ and responsible for nearly two-thirds of all global mortality with 41.1 million deaths in 2017.^[2]^ Cardiovascular disease (CVD) is 1 of the Non-communicable diseases and had become a major global public health problem. According to the Global Burden of Disease 2017 study,^[3–5]^ the worldwide numbers of CVD-related deaths increased from 11.9 million in 1990 to 17.8 million in 2017. An estimated mortality rate in 2017 was 233.1/100,000, in which 128.5/100,100 was in high-income countries, 254.1/100,000 was in upper-middle-income countries, 316.9/100,000 was in lower-middle-income countries and 285.3/100,000 was in low-income countries.^[2]^ Compared with high-income countries, the mortality rate of CVD was much higher in lower- and middle-income countries.

Income is 1 of the socio-economic indicators and there are mainly 3 ways expressing the relationships between income and health including the individual income, country's gross national product, and income inequalities among different areas.^[6]^ So far, numerous studies had indicated that income inequalities were a generalizable determinant that could directly influence some health outcomes,^[7]^ such as life expectancy,^[8–10]^ mortality,^[11,12]^ incidence,^[13,14]^ and so on.

As a common non-communicable disease, the health outcomes of CVD are also influenced by income. Researches found higher-income was associated with lower CVD mortality among the population aged 40 to 64.^[15]^ Moreover, associations between CVD and income were inconsistent among countries with different income levels. Taking heart problems as an example, in the high-income countries, the population aged 45 to 64 in the lowest income quartile had the highest risk of CVD (prevalence).^[16]^ But in the low- and middle-income countries, the results were contrary. The population aged 45 to 59 in the medium-income quartile had a higher risk than those in the lowest income quartile.^[17]^ In addition to age or countries, gender is also a factor which is clearly linked either with income and with CVD. For instance, negative associations between household income and hypertension prevalence were found in both genders. However, in the same level of income increase, the decrease in prevalence seemed to be more obvious in women than men.^[18]^ Therefore, generally speaking, there are still many unknowns in the field of “income and CVD” that deserve the attention of the academic community.

So far, there are many reviews articles have been published to summarize the associations between income and CVD, and discuss the status of CVD in countries with different income level.^[19,20]^ But it seems that no 1 has ever studied the current research dynamics of this field.

Bibliometric analysis is an effective method to quantitatively analyze the published scientific articles and illuminate the research trends, hotspots, and collaborations. It is fundamentally different from a meta-analysis. Meta-analysis is a statistical method that combines results from different studies and calculates a quantitative estimated overall effect of a specific intervention or variable on a particular outcome, and it is mostly used in clinical research. Unlike meta-analysis, bibliometrics analysis mainly focuses on the research dynamics of a specific study field and it has been applied in many disciplines, including medicine^[21]^ chemistry,^[22]^ computing,^[23]^ management,^[24]^ economic,^[25]^ and robotics.^[26]^

In this study, by using the bibliometrics analysis, we would like to present a different overview of this field with the following aspects:

(1) historical trend of the topic;
(2) the main contributor: leading countries, leading institutions, leading research areas and journals;
(3) representative authors;
(4) most frequently used keywords;
(5) most cited papers.

## 2 Methodology and data source

By using the Derwent Date Analyzer (DDA) software,^[26–31]^ the bibliometric analysis of this paper is based on publications related to “income and cardiovascular disease” published from 1990 to 2018. Literature was collected from the Science Citation Index-Expanded (SCI-E) and Social Science Citation Index on June 13, 2019. The document types were defined as review and article. The ethics approval of this study was not required because it did not use individual-level data.

CVD is a class of diseases that involve the blood vessels or heart, including coronary artery diseases, stroke, heart failure and so on.^[32]^ For developing a comprehensive search strategy for CVD, we searched the meta-analysis studies related to “CVD” in the Cochrane library. By integrating the search strategies of these studies,^[33–36]^ the search formula of CVD was finally formulated as TS = ((hyperlipid∗ OR hyperlip?emia∗ OR hypercholesterol∗ OR hypercholester?emia∗ OR hyperlipoprotein?emia∗ OR hypertriglycerid?emia∗) OR (“high blood pressure”) OR (hypertensi∗ OR “peripheral arter∗ disease∗”) OR (stroke OR stokes OR cerebrovasc∗ OR cerebral OR apoplexy OR (brain SAME accident∗) OR (brain SAME infarct∗)) OR (“atrial fibrillat∗” OR tachycardi∗ OR endocardi∗) OR (pericard∗ OR isch?em∗ OR emboli∗ OR arrhythmi∗ OR thrombo∗) OR (cardio∗ OR cardia∗ OR heart∗ OR coronary∗ OR angina∗ OR ventric∗ OR myocard∗)). Then, this formula of CVD and the formula of income (TS = income) were searched simultaneously in the databases of Web of Science (WoS). Since the “topic” searching in WoS is only applied to the title, abstract, and keyword, there are certainly some related publications have not been covered.

Using DDA software, search results were quantitatively and qualitatively analyzed. A line chart was used to describe the publishing trend of global countries. Bibliometric indicators including the number of citations, number of publications and *h*-index were showed in tables to characterize the “income and CVD” research from different aspects. A cross-relationship map was made to illustrate the collaborative relationships between countries/regions. A bubble chart was applied to show the development trends of journals and author keywords.

## 3 Results and discussion

### 3.1 The Performance of related publications and countries

**Figure 1:**
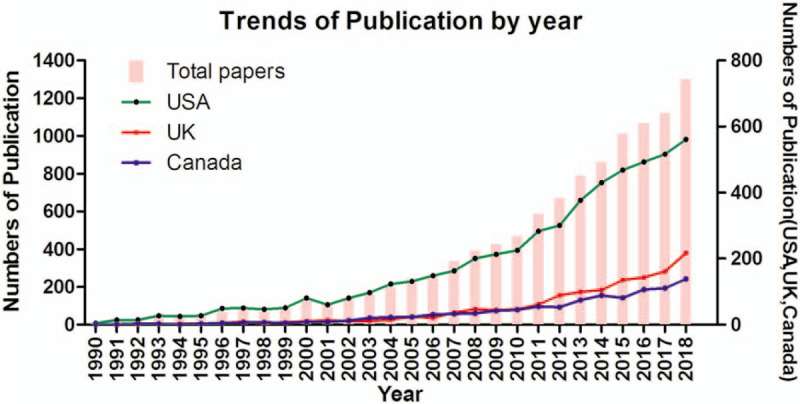
Trends in the total number of published papers related to income and CVD by year.

A total of 158 countries have published 17,764 articles and reviews, of which 264 are the Essential Science Indicators (ESI) Highly Cited articles and 13 are ESI hot articles. The total number of publications increased year by year (Fig. 1), which indicated that more and more scientists had focused their studies on this field. The top 3 most productive countries were the USA, UK, and Canada. The USA was the top country with most literature, and its increasing publishing trend was consistent with that of global. However, the increasing speed of the UK and Canada was relatively slower. The publications of these 2 countries started to show an obvious growth trend until 2002. During 2002-2011, they both increased almost at the same speed, but since 2012, UK exceeded Canada.

### 3.2 Cooperation of countries/regions

For better understanding, the contributions of each country/region in this field, the corresponding information of the top 20 most productive countries/regions were analyzed. As shown in Table 1, the USA was still at the top of the list and its publications had been accounted for almost half of the total papers. However, its indicator of average citations per paper (ACPP) was not the highest. In other words, the publishing quality of some countries (ie, Japan, Switzerland) was relatively better than that of the USA.

**Table 1:**
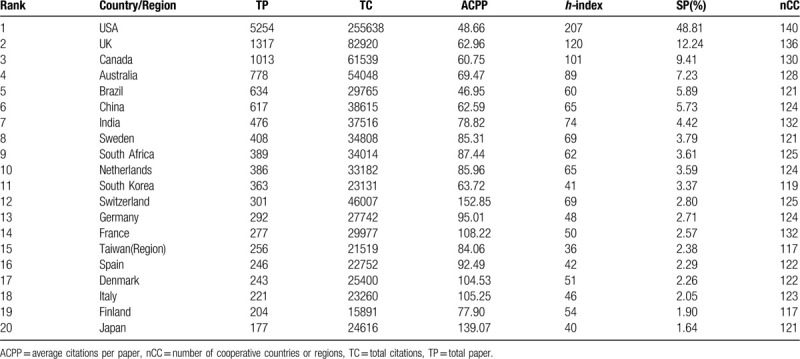
The top 20 most productive countries or regions in income and CVD field during 1990-2018.

Moreover, there were only 5 developing countries/regions in this top 20 list, including Brazil, China, India, South Africa and Taiwan (region). Compared with developing countries, developed countries were paying more attention to “income and CVD” research.

**Figure 2:**
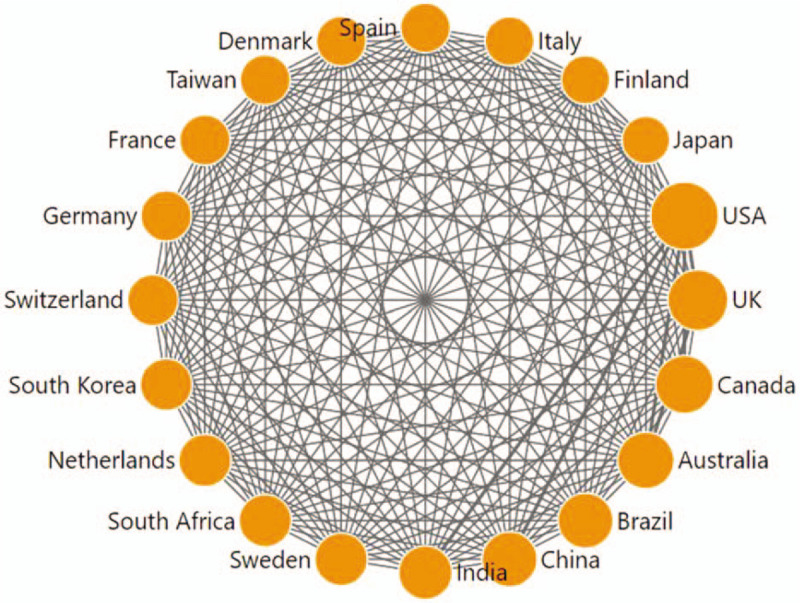
Collaboration matrix map among the top 20 productive countries/regions. Taiwan is a region of China.

Using DDA software, we draw a network diagram on the basis of the co-occurrence matrix (Fig. 2). The size of the node indicated the number of publications and the thickness of the connecting lines presented the frequencies of cooperation. As shown in Figure 2, there seemed to be no significant differences in cooperation between countries. Only the connecting lines between the USA and China, as well as the USA and India, seemed thicker than others. This might be indicated that developed countries like the USA had turned their researches into the income field of developing countries.

### 3.3 Contribution of leading institutions

The top 20 productive institutions in “income and CVD” research were shown in Table 2, along with their numbers of publications, citations, and *h*-index. Among the 20 institutions, the USA had the largest share (12), followed by the UK (2), Canada (1), Australia (1), Brazil (1), South Korea (1), South Africa (1) and Sweden (1). Obviously, the 20 institutions are from the 8 countries which were exactly in the list of the top 10 most productive countries (Table 1). Only 2 countries which were also in the list, China and India, had no institutions in the top 20. Coincidentally, China and India happened to be the countries with the closest cooperation with the USA, according to Figure 2. Thereby, scholars in China and India might publish their studies on “income and CVD” mainly through cooperation with USA institutions.

**Table 2:**
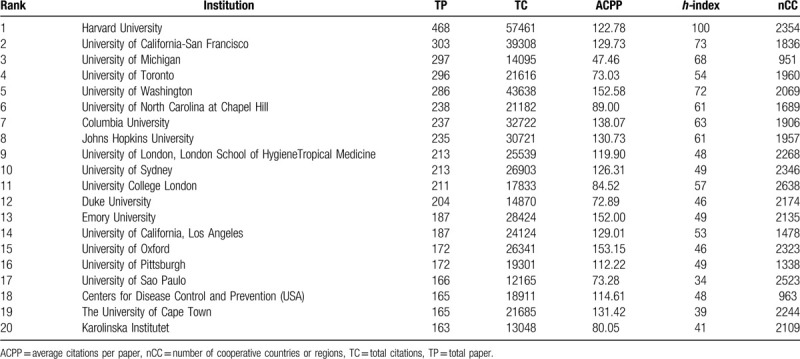
The top 20 most productive institutions of publication, citations and *h*-index during 1990-2018.

The top 3 productive institutions were also shown in Table 2, which were all from the USA. They were Harvard University, University of California-San Francisco and the University of Michigan in order. As for ACPP, the University of Oxford led the list with 153.15. Regarding the *h*-index, Harvard University still ranked first, also followed by the University of California-San Francisco and the University of Michigan, meaning the USA was an outstanding country with excellent academic institutions in this field of income and CVD.

### 3.4 Contribution of leading research areas and journals

The top 20 WoS research areas ranked by the number of publications related to income and CVD were shown in Table 3. Surprisingly, “public environmental occupational health” dominated the research area list with 3013 papers, followed by “cardiovascular system cardiology”, “general internal medicine” and “health care sciences services”. Among the top 20 areas, 18 of them were related to medicine and the other 2 were related to science technology or economics. From the list of top 20 areas, we also noticed that the study population in the “income and CVD” field was mainly focused on children, the elderly and the puerpera.

**Table 3:**
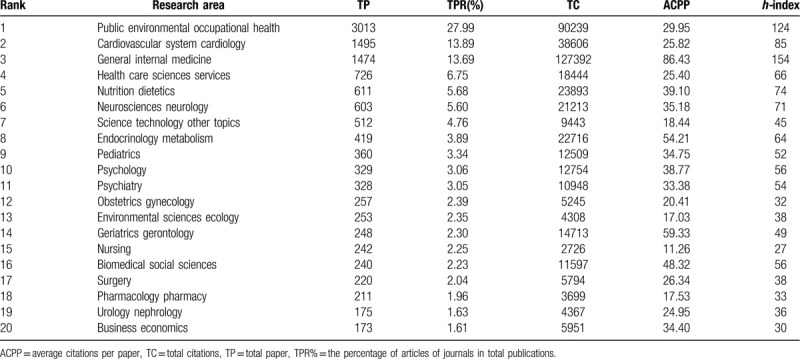
Contribution of the top 20 research areas in income and CVD research.

**Figure 3:**
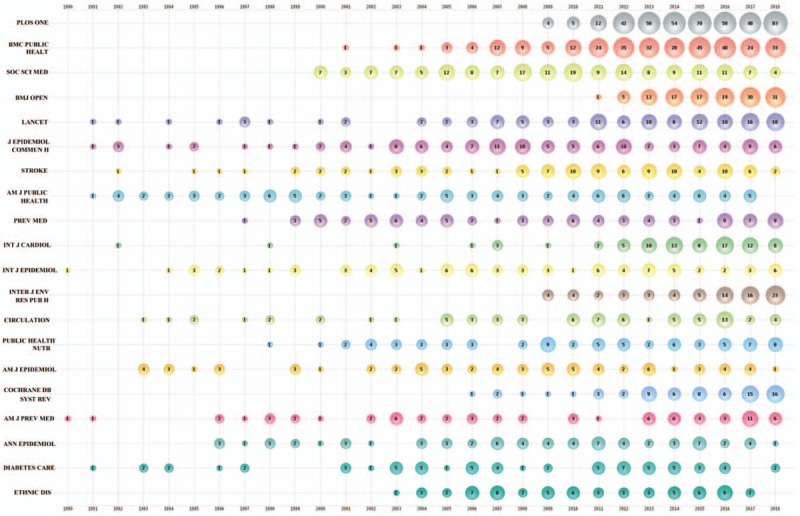
Bubble chart of top 20 productive journals by year.

The top 20 journals with most literature in the “income and CVD” field were listed in a bubble chart (Fig. 3). In 1990, Int J Epidemiol and Am J Prev Med firstly published related papers. The next year, in 1991, more and more journals began to publish papers related to income and CVD, including Lancet, J Epidemiol Community Health, Am J Public Health and Diabetes Care. As for the total publications during 1990-2018, Plos One took the leading position, followed by BMC Public Health, Soc Sci Med, BMJ Open and Lancet. But Plos One published the first paper since 2009. Both Plos One and BMC Public Health had seen a sharp increase in the number of publications since 2012. The annual publications of Soc Sci Med were relatively stable, but it seemed to have decreased in the past 2 years. Almost all these 20 journals were medicine related journals and the majority of them belong to the field of public health. In other words, current research on income and CVD mainly focuses on population prevention, rather than individual treatment.

### 3.5 Contribution of leading authors

The top 10 productive authors were shown in Table 4. According to the number of publications, Yusuf, Salim was ranked the first, followed by Lin, Herng-Ching and Dorairaj, Prabhakaran. As for ACPP, Ezzati, Majid led the list and the second place became Mensah, George A. Moreover, 5 of the top 10 authors came from developing countries, indicating scholars in developing countries had been paying attention to this field, although their national publications had not yet caught up with developed countries.

**Table 4:**
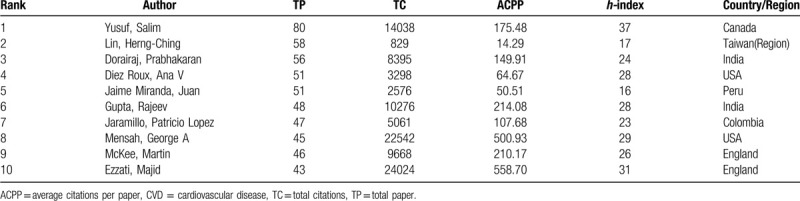
Contribution of the Top 10 Authors in Income and CVD Research.

### 3.6 Analysis of author keywords

To reveal the hot spots and development trend of income and CVD research; more than 12,000 author keywords were analyzed using DDA software. The keywords with the same meanings had been combined. However; since some publications do not require author keywords; these papers were not included in this analysis.

**Figure 4:**
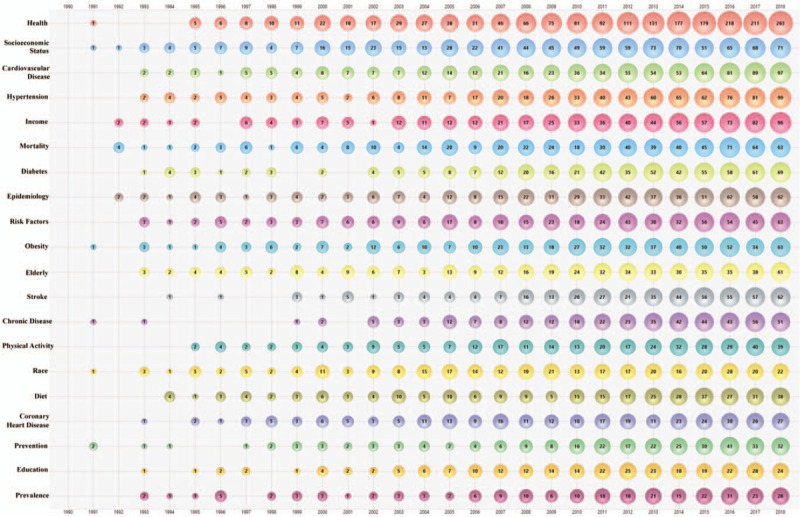
Bubble chart of top 20 author keywords by year.

The top 20 author keywords by year are shown in Figure 4. “Health^[37–39]^^”^ was the most frequently used keyword, then followed by “socioeconomic status,^[40–42]^^”^ “cardiovascular disease,^[43–45]^^”^ “hypertension^[46–48]^^”^ and “income.^[49–51]^^”^ The frequency of all the top 20 keywords increased during 1990-2018.

In this study, the keyword “Health” mostly referred to as “Health Disparity/Inequality.^[52–54]^^”^ A health disparity/inequality is a particular type of difference in health between different populations with various socio-demographic characteristics (i.e. socioeconomic status, racial/ethnic group, and education level).^[55,56]^ For instance, a health disparity of coronary heart disease was observed in Americans with different family incomes and races. The worthiest Americans and Hispanics had the least risk of coronary heart disease.^[49]^ Therefore, it was obvious that the published papers in this field mainly focused on evaluating the health disparity of CVD among different income levels.

Moreover, studying on these top 20 keywords, we could also find some hot spots in “income and CVD” field. First, the elderly^[57–59]^ were the main study population in this field. Its main concern was the impact of income on CVD risk,^[60]^ the efficacy of CVD therapy,^[61]^ and the CVD outcome^[62]^ in the elderly. Second, besides “income”, “race^[57,63,64]^^”^ and “education^[65–67]^^”^ were the most frequent social-demographic characteristics. In terms of either racial disparity or differences in education levels, the disease burden of CVD was different. Black persons and White persons lost 1.2 and 0.1 potential life-years before 75 years of age per person, respectively, with a difference of 1.1 years. Compared with the more-educated persons (2.8 potential life-years), the less-educated persons lost more potential life-years (4.6) for CVD.^[67]^ Third, “disease prevention”^[68–70]^ (including primary prevention^[71]^ and secondary prevention^[72,73]^), and “risk factor”^[68,74,75]^ were the main study direction. For instance, socioeconomic status, childhood and early-life factors, inequalities in health services, place of residence, wealth distribution, and work-related factors can affect CVD risk. Medical workers should reduce these risk factors via effective primary and secondary prevention.^[71]^ Forth, “prevalence”^[76–78]^ and “mortality”^[38,79,80]^ were both the study epidemiological indicators of this field, including hypertension prevalence,^[81]^ prevalence of peripheral artery disease,^[82]^ cardiorespiratory mortality,^[83]^ premature cardiovascular mortality^[84]^ and so on. Fifth, the most concerned health issues in this field were “obesity”^[85–87]^ and “diet”^[85,88,89]^. Income may affect someone's dietary intake behaviors, leading to obesity which was 1 of the risk factors for CVD. Sixth, hypertension,^[90,91]^ diabetes,^[92–94]^ stroke^[95–97]^ and coronary heart disease^[98–100]^ were the most studied chronic diseases associated with this field.

Moreover, gender is a factor which is clearly linked either with income and with CVD. Therefore, the keyword “gender” was studied although its frequency was not in Top 20 (ranked 33rd). First, women were more concerned than men about the relationship between income and CVD, such as low-income elderly women,^[101]^ women at high risk for stroke,^[102]^ black women^[103]^ and so on. Second, “gender-differences” were the most discussed topics, and low income seemed to have a greater impact on the CVD risk in men than women. For instance, compared with women, 10-year estimated coronary heart disease risk was more tightly linked in men with lower household monthly income.^[104]^ Also, low income was associated with hypertension prevalence in men, but not in women.^[105]^

### 3.7 An analysis of the most cited papers

The number of citations is an indicator that evaluating the impact of a scientific publication, and it reflects an article's contribution to the field of interest. Many scholars had published bibliometric analyses on the most-cited papers in a specialty field.^[106–109]^ In this study, we also used this indicator of an article to reflect the hotspots of “income and CVD” research. The top 10 most cited publications were listed in Table 5.

**Table 5:**
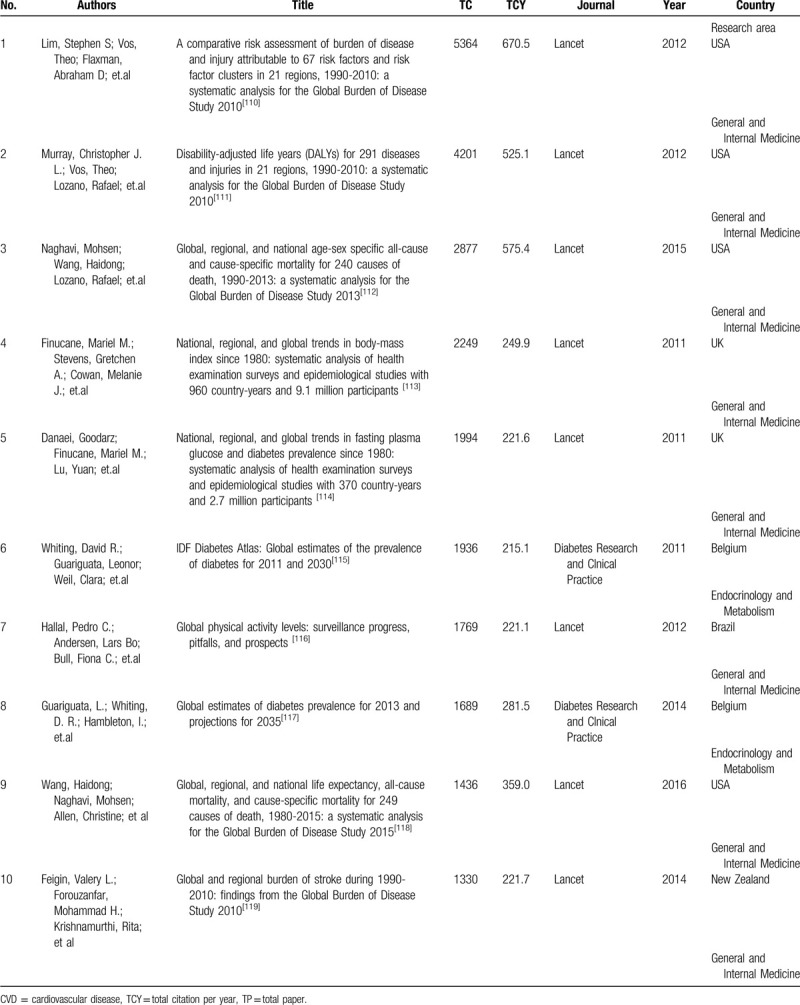
Top 10 most cited publications in income and CVD research during 1990-2018.

The most highly cited paper was “A comparative risk assessment of burden of disease and injury attributable to 67 risk factors and risk factor clusters in 21 regions, 1990-2010: a systematic analysis for the Global Burden of Disease Study 2010” published in Lancet. This paper described the distributions of risk factors (ie, high blood pressure) in countries with different income levels.

The contents of the other 9 most cited papers were basically similar to this 1. They were all descriptive studies of global health issues using epidemiological indicators and could help scholars understand the global health issues quickly and accurately. Therefore, they became the top 10 most cited papers.

However, these 10 most cited papers might not be the best articles explaining the associations between income and CVD. Articles that explore the associations between income and CVD with a global perspective are urgently warranted.

## 4 Conclusions

Based on bibliometrics and DDA software, this study provides a new perspective on the field of “income and CVD” through the information analysis involving yearly publication trends, leading countries, institutions, authors, journals, keywords and so on. Several findings could be summarized as follows.

First, the field of “income and CVD” has attracted the increasing attention of numerous global scholars.

Second, compared with developing countries, developed countries are paying more attention to “income and CVD” research. As developing countries, China and India have the closest cooperation with the USA, and these 2 countries may publish their studies on “income and CVD” mainly through cooperation with USA institutions.

Third, the elderly, the children and the puerpera are the main study population in this field, especially for these populations of different races and educational levels.

Forth, “disease prevention” is the main study direction, and “prevalence” and “mortality” are the main study epidemiological indicators of this field.

Fifth, the most concerned health issues in this field are “obesity” and “diet”, and the most studied chronic diseases are hypertension, diabetes, stroke, and coronary heart disease.

Sixth, there is a lack of articles that explore the associations between income and CVD with a global perspective. Articles on this content are urgently warranted.

In general, this study can help scholars better understanding the global overview of “income and CVD”, and provide the information of potential collaborators, as well as the information of promising attractive areas for future research.

## Author contributions

**Conceptualization:** Yuehua Wan, Qing Shen.

**Data curation:** Xufen Ding.

**Formal analysis:** Xufen Ding, Yuehua Wan.

**Funding acquisition:** Ye Ding, Dingwan Chen.

**Investigation:** Ye Ding, Dingwan Chen, Xufen Ding, Guan Wang

**Methodology:** Qing Shen.

**Project administration:** Yuehua Wan, Qing Shen.

**Resources:** Dingwan Chen.

**Visualization:** Guan Wang.

**Writing – original draft:** Ye Ding.

**Writing – review and editing:** Yuehua Wan
